# The social ecology of adolescent mental health in humanitarian settings: A qualitative study

**DOI:** 10.1371/journal.pmen.0000543

**Published:** 2026-01-22

**Authors:** Sally Farah, Tania Bosqui, Anas Mayya, Zahraa Shaito, Joseph Elias, Theresa S. Betancourt, Alan Carr, Michael Donnelly, Felicity L. Brown

**Affiliations:** 1 Department of Psychology, American University of Beirut, Beirut, Lebanon; 2 Trinity Centre for Global Health, Trinity College Dublin, Dublin, Republic of Ireland; 3 Research and Development Department, War Child Alliance, Amsterdam, The Netherlands; 4 Boston College School of Social Work, Chestnut Hill, Massachusetts, United States of America; 5 School of Psychology, University College Dublin, Belfield, Dublin, Republic of Ireland; 6 Centre for Public Health, School of Medicine, Queen’s University Belfast, Belfast, Northern Ireland, United Kingdom; 7 Amsterdam Institute of Social Science Research, University of Amsterdam, Amsterdam, The Netherlands; National Psychological Association of Ukraine, UKRAINE

## Abstract

The social-ecological context of adolescent mental health, particularly adolescents living through humanitarian crises, is understudied. There is a pressing need for such research to inform the development of contextualised systemic interventions for this population. This study investigated the social ecological stressors and risk- and protective-factors experienced by adolescents and their families in crisis-hit Lebanon to inform a contextually grounded systemic psychosocial support intervention. A whole-family systems approach grounded in the Social-Ecological Model was used with a qualitative design to capture the lived experiences of adolescents. Interviews were conducted with 46 Lebanese and Syrian adolescents (48% female, mean age = 13.8 years, SD = 1.65), their families (*N* = 42 caregivers, 60% mothers), and experienced providers (*N* = 5) in Lebanon. Transcripts were analysed using Reflexive Thematic Analysis which highlighted six interconnected themes (25 sub-themes) that characterised the life experience of adolescents at each identified systems level. Themes ranged from adolescent mental health and interpersonal relationships to living conditions and access to basic human rights and services. Gender and nationality exerted cross-cutting influences across themes. Findings are discussed drawing on social ecology, systems theory, and liberation psychology. Results highlight a clear need for interventions to involve families and caregivers, and consider adolescents in their social ecological contexts. This approach could better address the needs of adolescents, by supporting adolescent development, enhancing mental health and well-being, reducing community and social isolation, and, more broadly, advocating for social equity, justice, and human rights.

## Introduction

Adolescence is a formative period of development that marks the peak onset of mental health disorders [1]. The increasing prevalence of mental health disorders globally [2,3] has made such conditions the leading cause of disability in young people [4] with half of all mental disorders emerging before age 18 [5]. Social determinants of mental health like poverty, social disadvantage, exposure to community or family violence, and armed conflict are all strongly related to this high prevalence [6]. In conflict-affected and humanitarian settings, 1 in 5 people meet the criteria for a mental disorder [7]. Within humanitarian settings affected by protracted and multi-layered crises, about two-thirds of adolescents have a probable disorder [8,9], demonstrating the impact of chronic collective stress exposure.

Systems theory has long advocated for the inclusion of the social ecological and collective experience in our understanding of mental health, particularly for populations experiencing oppression, injustice, and inequity. Liberation Psychology [10] theorises that treating mental disorders as individual afflictions unrelated to societal oppression perpetuates this harm. Instead, it advocates for active acknowledgement and the development of a ‘critical consciousness’ to address root causes of mental distress and promote social change. Such theories have been strongly supported by research since, with social determinants clearly and consistently related to mental health, and with the most oppressed and socio-economically disadvantaged disproportionately diagnosed with mental disorders, as well as involuntarily hospitalised [11,12]. Social determinants include each systems level of the Social-Ecological Model [2,13]; the immediate family and social environment (Microsystem), the community and neighbourhood (Exosystem) and the socio-economic and cultural setting (Macrosystem), as well as interconnected feedback loops between them.

Within the microsystem, family stress, poor communication, and conflicts in the family are strongly associated with poorer adolescent mental health [9,14], as well as insecure attachment styles, poor parental mental health, and punitive parenting styles [15,16]. In the exosystem, community-level determinants of adolescent mental health include neighbourhood disadvantage and poor community cohesion [17]. In the context of war, collective experiences have been associated with high individual anxiety [18], demonstrating the relationship between the collective and the individual. At the macrosystem level, socioeconomic insecurity, unemployment, poverty, and food insecurity have all been associated with poorer mental health of both parents and adolescents [19–21], as well as harmful gender norms, child labour, early marriage [22–24], racism and discrimination [25], and poor access to healthcare and education [26–29].

Despite such clear and consistent evidence for the role of social determinants in explaining high levels of distress for at-risk adolescents living in humanitarian settings, the social ecological system is rarely centred within mental health and psychosocial support (MHPSS), and there is often little exploration of how stressors at different levels of the social ecology compound and interact with one another. As a result, very few MHPSS interventions focus on the systems around the child, consider the mutual interconnections between the adolescent, family, community, and society, or account for the subsequent and bidirectional impacts on adolescent distress. A review of systemic interventions in low- and middle-income countries [30,31] found that few interventions targeted humanitarian settings, particularly in the Middle East and North Africa (MENA) region, and adolescents, demonstrating a critical gap in the literature.

Recognizing the critical need to investigate the social deteminants of mental health in humanitarian settings in the MENA region, we conducted a qualitative enquiry with adolescents and their families living in adversity in the crisis-hit country, Lebanon. Guided by Brofenbrenner’s ecological systems theory [13] and family systems theory, we aimed to explore stressors and risk and protective factors across each level of the adolescent’s social ecology, and the interactions between them. This study is part of a larger study aiming to develop and test a family systemic psychosocial support intervention for humanitarian settings in the MENA. The research questions that were answered were; a) what are the individual, family, community, and systems influences on adolescent and caregiver mental health? and b) in what ways do adolescents and caregivers cope?

## Methods

### Design

A qualitative design was used to explore adolescent and caregiver mental health using a whole-family systems approach, with interviews stuctured by the social-ecological model’s different systems levels [13]. With approval from the American University of Beirut Institutional Review Board, exploratory interviews were conducted with the whole family unit and separate interviews with each adolescent and caregiving family member in the household, as well as follow-up interviews as themes emerged from the data.

### Setting

Lebanon, a lower middle-income country affected by a devastating financial collapse and longstanding refugee crisis, is one of the largest hosts of refugees but also suffers one of the world’s worst economic crises. This has plunged the majority of the population into poverty, and refugees further into crisis – over 90% of Syrian refugees need humanitarian assistance to meet basic needs [32]. The population residing in Lebanon also suffered through the Beirut port explosions on August 4, 2020, displacing hundreds of thousands of people. Fears for the long-term impact of multiple and protracted crises on mental health are well founded, leading to a call to action to pre-empt Lebanon’s ‘silent epidemic’ [33]. This is ever more pressing after over two years of war since October 2023, that has killed or injured 15,000 people and displaced over one million [34].

### Sample

Participants were recruited using a maximum variation sampling strategy aiming for equal numbers of male and female, younger (12–14) and older (15–17) adolescents, with different nationalities (Lebanese, Syrian, Palestinian). This age group was selected based on an identified intervention gap for this age, as this formative study was used to inform intervention development. In total, the sample population consisted of 26 families living in the Beddawi urban area of North Lebanon (adolescents *n* = 45, caregivers *n* = 42) and five practitioners (child protection case managers *n* = 3, MHPSS facilitators *n* = 2) experienced in working with adolescents in Lebanon.

Eligibility criteria for families included: a) having an adolescent between the ages of 12 and 17 years, b) at least one caregiver aged 18 years or older, and c) adolescent assent and caregiver consent. Exclusion criteria were: a) signs of significant cognitive impairment due to a severe psychiatric disorder or other condition that would prevent adolescents from participating meaningfully in the interview, or b) either the adolescent not assenting or the caregiver not consenting. Inclusion criteria for the practitioners included a) two or more years of MHPSS experience with children, adolescents and caregivers in Lebanon, and b) written consent.

### Procedure

Staff from OMITTED, an international non-governmental organisation specialising in MHPSS and child protection, contacted potential participating families. Outreach workers, independent of the study, contacted families either via telephone or in-person, through OMITTED case management service or the OMITTED Centre. Meetings with interested and eligible families were held in the OMITTED Centre in Beddawi, local to families’ homes. Where in-person interviews were not possible, they were conducted over the phone (n = 11 families). Compensation of 10,000 LBP (worth 6.6 USD officially but with an actual street value of 1.9 USD due to the economic collapse at the time of interviews) per family was provided to cover transportation or call costs. The level of compensation was based on the need to compensate for participants’ time and travel expenses without coercing them into participation, which was a major challenge given the economic collapse and widespread poverty.

Written consent was obtained from caregivers, and assent was obtained from adolescents. Then, interviews were conducted using a whole family approach. At the end of each family interview, every family member was interviewed individually. This was to counteract any family and group dynamics that may have made it challenging for all views to be expressed freely, such as for adolescents in front of their parents. Three families were unreachable for follow-up. All interviews were conducted between July 13, 2020, and November 27, 2020. It is important to note that the Beirut port explosion occurred on August 4, 2020, during data collection. Increased mental health difficulties were understandably noted after the blast [35], particularly for those directly affected [8]. The aftermath of the explosion affected both participants and the research team.

Semi-structured interviews lasted between 90 and 120 minutes and were conducted in Arabic by graduate level research assistants (RAs) trained in project-specific qualitative interviewing, including role play and feedback, by co-Principal Investigators (PIs) TB and FB. Although we had initially decided to recruit and interview 15 families, later data collection unveiled new information. During a preliminary validation workshop with our Community Advisory Board (CAB), it was raised that we had not explored girls' labor, as it was largely unpaid and within the home. We therefore met with additional families to explore this gap.

The interview guide was structured around the social-ecological model [13] and included questions about strengths and difficulties at each system level; the Macrosystem socio-political context, the Exosystem community level, the Microsystem family level, the Individual adolescent and parent levels, and interactions between systems. Using a whole-family systems approach also allowed experiential observation of dynamics within families. Questions were kept open and broad to allow for exploration. Member checking through follow-up interviews, community consultation, and duplicate coding, as well as triangulation through inclusion of adolescents, caregivers, and service providers, were conducted to strengthen the validity of findings.

All interviews were audio-recorded with consent and de-identified. They were transcribed and translated into English by the RAs, study coordinator, and student volunteers. Transcriptions and translations were quality-checked by the Study Coordinator ZS before analysis. Detailed field notes taken during interviews and reflection diaries were also kept by RAs.

### Ethics statement

The present study has been approved by the American University of Beirut Institutional Review Board and it complies with ethical standards outlined in the Belmont Report. As mentioned, ethical approval was obtained from OMITTED. Also, written informed assent was obtained from adolescents, and written informed consent was obtained from their legal guardian(s).

#### Inclusivity in global research.

Additional information regarding the ethical, cultural, and scientific considerations specific to inclusivity in global research is included in the Supporting Information (S1 Checklist).

#### Positionality statement.

The core research team were Lebanon based, with interviews conducted by Research Assistants including one Lebanese female (SF) and one Syrian male (AM) and supervised by one British/French (TB) and one Australian (FB) Lebanon-based clinical psychologists. Whilst we had extensive experience and understanding of the local context, we were also from far more privileged backgrounds than our participant population, particularly in terms of financial security and educational opportunity. We therefore maintained reflective diaries and debriefing conversations between interviews to question and challenge unhelpful assumptions or challenges in meaningfully reflecting our participants experiences.

### Analysis

Data analysis was carried out using inductive and deductive Reflexive Thematic Analysis [36] using axial coding. Data analysis was conducted by SF and AM who had also carried out the interviews. In the first step of thematic analysis, SF and AM reviewed the transcripts to re-familiarise themselves with the content. Secondly, the transcripts were uploaded to MaxQDA (version 2020) and open coded by SF to identify patterns of data segments that were perceived relevant to the proposed research questions. Data segments in the form of direct quotes were coded. For validity and reliability, the first 35% of transcripts were independently coded by AM, discrepancies were discussed, and a consensus was reached. To ensure optimal fit with the data, codes were collapsed or clustered. In the third step, the team (SF, AM, TB, and FB) constructed and defined reoccurring categories and then applied codes to the relevant categories. In the fourth step, axial coding was carried out to explore the relationship within and between categories. Organised according to the social-ecological systems levels [13], categories were initially grouped into 14 themes and 76 subthemes. After review, they were refined and collapsed into six themes and 25 subthemes that described the most prominent patterns in the data. In the fifth step, to examine the validity of the data, the local CABs reviewed a summary of the findings, suggested alternative interpretations, and discussed potential implications of each theme’s core component. The themes were subsequently edited based on the feedback.

In the sixth step, reflections and analytic memos were compiled by TB including reflective diaries, field notes, and meeting minutes, which were then grouped and organized to inform the interpretation of interview data. This is in line with the approach [37] to rigorous reflexive inductive qualitative inquiry. In the final step, findings were reported using the Consolidated Criteria for Reporting Qualitative Studies (COREQ) guidelines [38].

All data, analysis code, and research materials are available upon written request to the corresponding author. This study’s design and its analyses were not pre-registered.

## Results

### Participants

A total of 46 adolescents and 42 caregivers from 26 families participated in interviews. As shown in Table 1, mothers were present in almost all the interviews (96%) while fathers were present in 17 out of 26 (65%) interviews. Forty-eight percent of the adolescents were female, and ages ranged between 12 and 17 years, with a mean age of 13.8 years (SD = 1.65). Of the 26 families, 16 (62%) were Syrian and 10 (38%) were Lebanese. We were not able to recruit any Palestinian families. All families resided in Beddawi, Tripoli, with eight of them residing inside the Beddawi Refugee Camp. 18 families were recruited through case management while either were recruited by outreach. In addition to the family interviews, three case managers participated in interviews at the OMITTED office in Mina, Tripoli, and two MHPSS officers participated in interviews via telephone.

**Table 1:** Participant characteristics.

**Participants**	n
Adolescents	46
Caregivers	42
Facilitators	5
**Caregiver attendance**	n(%)
Mothers	25 (96%)
Fathers	17 (65%)
**Adolescent Sex**	n(%)
Female	22 (48%)
Male	24 (52%)
**Adolescent Age**	m(SD)
	13.89 (1.65)
**Family Nationality**	n(%)
Lebanese	10 (38%)
Syrian	16 (62%)

### Themes

**Fig 1:**
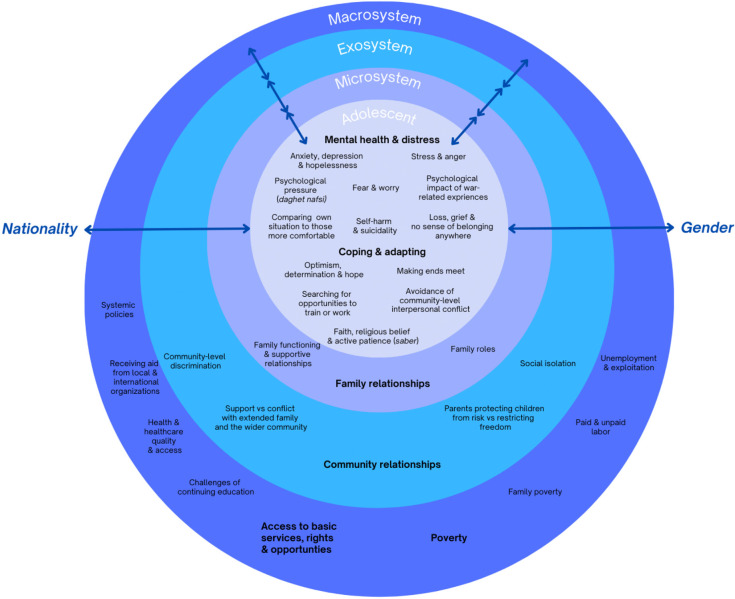
Systems-Levels, Themes, Subthemes, and Cross-Cutting Influences Mapped onto the Social-Ecological Model.

Six overall themes with 25 subthemes were identified across four inter-linking nested systems levels with cross-cutting influencing factors of gender and nationality. Themes reflect stressors and coping mechanisms at each systems level which impact adolescent mental health and functioning described at the individual systems level. The social-ecological model begins at the outermost Macrosystem, encompassing broad systemic influences on adolescent mental health and wellbeing including access to basic rights, services, and opportunities, and poverty. Moving inward, the Exosystem includes indirect interactional relationships, such as with the neighbourhood and community, that shape the adolescent’s environment. The Microsystem comprises immediate contexts such as the adolescent’s relationship with family members. Importantly, bidirectional interactions exist across all levels centering on the Adolescent at the core. Within this core, adolescent mental health and distress, and adolescent coping and adapting were explored. The themes within each nested systems level are visually illustrated in Fig 1, comprehensively listed in Table 2, and described below.

**Table 2:** Systems-levels, themes, and subthemes.

Systems Level	Theme	Subtheme
Macrosystem		
	Access to basic services, rights, and opportunities	
		Challenges of continuing education
		Health and healthcare quality and access
		Receiving aid from local and international organizations
		Systemic policies
	Poverty	
		Family poverty
		Paid and unpaid labour
		Unemployment and exploitation
Exosystem		
	Community relationships	
		Support v. conflict with extended family and the wider community
		Community-level discrimination
		Parents protecting children from risk v. restricting freedom
		Social isolation
Microsystem		
	Family relationships	
		Family functioning and supportive relationships
		Family roles
Adolescent		
	Mental health and distress	
		Anxiety, depression, and hopelessness
		Psychological pressure (*daghet nafsi*)
		Comparing own situation to those more comfortable
		Fear, worry, stress, and anger
		Self-harm and suicidality
		Psychological impact of war-related experiences
		Loss, grief, and no sense of belonging anywhere
	Coping and adapting	
		Optimism, determination, and hope
		Searching for opportunities to train or work
		Making ends meet
		Avoidance of community-level interpersonal conflict
		Faith, religious belief, and active patience (*saber*)

The social-ecological model starts at the outermost (A) Macrosystem level and progresses inward through the (B) Exosystem level and (C) Microsystem level, which encompass community and family environments, respectively. Bidirectional interactions across the four nested systems levels converge on the (D) Adolescent at the centre of the concentric circles.

### Macrosystem

The Macrosystem level encompasses broad systemic, societal, and cultural forces as well as essential services and economic conditions that form the outermost layer influencing adolescent mental health within Bronfenbrenner’s ecological framework. These overarching structures set the contextual backdrop for more proximal influences, with bidirectional effects shaping adolescents’ experiences and wellbeing.

#### Access to basic rights, services, and opportunities.

At the Macrosystem level, adolescents and their parents faced major barriers to accessing basic services, rights, and opportunities. Firstly, several obstacles impeded adolescents’ access to education, with poverty being paramount. It shifted parents’ priorities and focus towards ensuring their children’s basic needs and survival. Parents often could not afford tuition or safe transportation, and in many cases, adolescents were required to drop out of school to engage in paid labour or childcare at home. At the societal level, parents had safety concerns when sending adolescents, particularly girls, to school due to the prevalence of harassment at school gates. One mother said, *‘[my child] stopped going to school because there were no buses, so we started to fear for her…Men were sexually harassing her a lot at the door of the school, trying to kidnap her.’* At the institutional level, schools implemented a double-shift system to deal with overcrowding, with evening classes allocated to Syrian students. This system reduced the number of hours Syrian children spent in school and made it increasingly difficult for them to attend due to exacerbating safety concerns at night. Still, even Syrian students who overcame these obstacles and obtained access to an education faced additional challenges with a drastic change in language or dialect of instruction, and differences in curriculum. Moreover, the onset of the COVID-19 pandemic brought additional barriers for some families due to a lack of access to devices and internet connection required to participate in remote classes. These compounding barriers significantly impeded adolescent access to education.

In terms of healthcare access, families experienced significant health problems and emphasized their inability to afford essential medical treatment, including preventative medicine. One mother shared, *‘If he (child) gets sick, I can’t treat him...he didn’t receive his vaccines, and I can’t afford it (chokes in tears).’* Participants reported that where healthcare was available, it was generally of low quality, with limited clinics providing free or affordable healthcare.

The crippling economic and political crisis in Lebanon, with the Lebanese Lira losing over 98% of its value as a result of a lack of functioning government and widespread corruption, brought about overwhelming challenges for families and hindered their ability to meet their basic needs. The shortage of government-supplied services, including water and electricity, deprived families from minimum living standards. Compounding this, systemic discriminatory policies and practices perpetuated this cycle for Syrian refugees by impeding their freedom of movement, work and education opportunities, and access to basic services. A Syrian father reflected, *‘I may be walking on the street, and they ask me for my papers…they detain me for three days…it makes working very difficult too. One may go to work or be taken by a checkpoint.’* Although the civil society sector played a fundamental role in providing financial assistance and mental health services, with families highlighting the support they received from local, international, governmental, and non-governmental organizations, there was a consensus that the demand substantially outweighed available resources. One Syrian mother stated, *‘They’ve helped me with shared housing. I gave birth through help from the UN…but maybe now with the large amount of people, they don’t have the manpower and resources to handle it.’*

#### Poverty.

In parallel to the Macrosystem level was the intersection between access to rights, services, and opportunities, and poverty and child labour. Not only was poverty an obstacle to families’ basic rights to education and healthcare, but it also served as a barrier to the right to an adequate standard of living. Families reported that they struggled to secure a livelihood. They shared their experiences of living in poor housing conditions, being at risk of eviction, and not being able to provide enough food for their children. One Syrian mother explained, *‘The children want to have dinner, and the food was not enough. The children slept hungry.’*

Parents emphasized that this cycle of poverty was perpetuated by adult unemployment and exploitation which was particularly faced by Syrian parents due to experiences of discrimination and legal restrictions for the employment of Syrian people. This has seen the emergence of an informal and unregulated market for labour.

As a result of financial hardships and adult unemployment, many adolescents were the breadwinners in their family. Child labour took many forms and children worked long hours with minimal compensation. One 12-year-old Syrian adolescent described feeling exploited at work, *‘We used to go out from eight till two o’clock. I would come back having only made 4,000 [LBP, worth less than 0.5 USD at the time of the interview] and feeling sad. What do they provide me with those 4,000?!’* Additionally, adolescents reported being exposed to violence both at work and on their way to and from work. When adolescents, particularly girls, were not participating in labour outside the home, they held responsibilities for domestic work in the home, such as taking care of younger siblings and household chores.

### Exosystem

The Exosystem level comprises indirect environmental settings around the individual and their Microsystem. Themes at this level highlight how community dynamics interact with broader societal forces to affect individual mental health experiences.

#### Community relations and support.

One major theme within the Exosystem revolved around community relations and support. While some community members - including extended family members, friends, and neighbours - supported one another amid their daily struggles, others faced conflicts with one another, as a Syrian mother shared about her Lebanese neighbour, *‘Yesterday our neighbour attacked my husband and if he had said another word, she would have probably hit/killed him in the middle of the road’.* Families highlighted a lack of trust and ‘*collective depression’* within the community, as one Lebanese father described, ‘*Everyone is living this collective depression and these problems that we are facing*’. In addition to the lack of trust and conflicts with other members of the community, families faced abuse from authority figures within the community. MHPSS facilitators and families reported cases of exploitation and manipulation, including unpaid labour and manipulation by unlicensed doctors. They also explained that in some cases, unofficial community authorities (*Shawish*) and religious figures exerted their power and control over the community for monetary benefits, as one case manager explained, *‘In the [refugee] camps, the Shawish [unofficial community authority] is involved in everything…he tells them [families] that they have to work in this…or he threatens to kick them out.’*

Families described the dangerous conditions of their neighbourhoods. Given scarce financial resources, families would move to cheaper neighbourhoods, to pay relatively lower rent prices which usually came at the cost of living in more dangerous areas. Caregivers spoke about negative influences on the streets including smoking and the use of drugs and alcohol. Many families described their neighbours and landlords as being violent and disrespectful. One Syrian mother illustrated, *‘They keep shouting and uttering obscenities. At 3am there’s this guy over there who gets drunk and [takes] drugs. He keeps hitting his mother and the kids are scared of him at night. Our house is close to his.’*

Compounding their hardships and, for Syrian refugees, prior traumatic experiences from the war, families detailed community reports and personal experiences of kidnap, physical assault, and sexual harassment and assault. They witnessed thefts, beatings, stabbings, and shootings. Additionally, Syrian families described being subject to discrimination and competing over limited resources and living space. They received threats and were subject to verbal abuse and violence regularly, as one Syrian mother shared*,’Syrians, you do not have anything here, go back to your country [quoting another person]…if we are walking on the street, they spit at us...the dog is more valued than us.’*

To protect their children in unsafe neighbourhoods, parents restricted their time out of the house. Parents took stricter measures when it came to ensuring their daughters’ safety. They made sure their daughters were accompanied by someone whenever they left the house. In many families, both adult and adolescent women reported not being allowed to leave the house to socialize or work to avoid gossip from society members and to keep their family’s honour intact, as a Lebanese mother explained, *‘She [daughter] likes to interact with a lot of people and her father won’t be offended like this…they talk about you…The girl, if she is like this, nobody will marry her…When she goes out of the house, she is ruined.’* In some cases, families reported that protecting the family’s honour was achieved by arranging a marriage, which in many cases occurred during adolescence, causing distress among adolescent girls in particular.

### Microsystem

The Microsystem level represents immediate, direct settings, including familial relationships, within which adolescents engage daily and experience bidirectional influences on their mental health. This proximal layer can mediate effects from outer systems, revealing how family interactions serve as a critical buffer for adolescent wellbeing.

#### Family functioning and relationships.

Within the microsystem emerged the interrelatedness of family members, their roles, functioning, and relationships which were linked to stress and coping. On the one hand, many participants described their family as being their main source of strength during challenging times. They sought help from and opened up to one another. On the other hand, many family members spoke about not receiving support from their direct family members and many preferred to keep their feelings and thoughts to themselves, partly to avoid burdening others with their worries.

Mothers commonly took on the majority of housework and childcare, even when fathers were unemployed. One facilitator described, *‘Unfortunately, many of the men considered the responsibility of raising the children rested on the women… there was this clash between the couples; like no, the mother is the one that raises the children, not me.’* Fathers aimed to fill the traditional family breadwinner role. Their inability to do so instigated distress and frustration, which they freely expressed. However, mothers described feeling required to keep their composure for the overall well-being of their family despite their struggles and worries. Many mothers took on a peace-making protective role in their families. They described trying to keep both their husbands and children calm and content and protected their children from their father during conflicts. One Syrian mother shared, *‘In the house you see me as everything; trying to calm that down and being understanding when that one is angry…as a mother, I soothe things for them, make it look easier than it is…pressure everywhere…if you talk to my husband, he would lose his temper at any word you say to him.’*

Parents described their children’s childhood as being lost, as one Syrian mother reflected, *‘They have been denied their childhood in the first place… [my son] goes out and works…[my daughter] seems as if she is an old homemaker…as children, they should be living their childhood and they should be learning.’*

Family members took various measures and adjusted usual family roles to support the family and relieve the burden on others. Adolescents took on work responsibilities as they felt the pressure from their parents. A 12-year-old Syrian adolescent shared*, ‘I don’t like to work, but I like to help my parents so they’re not under pressure. [I feel] pressure, when it comes to money yes…there’s pressure on the household; we don’t have money.’* Parents tried to shield their children from the family’s financial troubles, including depriving themselves of food, or sourcing food and clothing through different means, as one Syrian mother shared, *‘I avoid letting my kids know that I can’t afford to buy them clothes…I make sure not to make them feel as if I am getting them worn clothes from the streets*.’

### Adolescent

At the core of the ecological model lies the Individual level, centering on adolescents’ emotional, cognitive, and behavioural experiences of mental health and coping, which both respond to and actively shape influences from surrounding systems. This innermost layer, nested within the three outer systems levels, integrates all prior themes into personalized experiences of both distress and resilience.

#### Mental health and distress.

At the Individual level, the experience of stress and distress emerged within the context of the wider social ecology, at every systems level. Stressors at the family and society level impacted family members at the individual level in several ways. Mothers felt overwhelming psychological pressure (or *daghet nafsi* in Arabic) while taking on the role of the peacemaker in the family. According to mothers, fathers were expected to be the breadwinners of the family at the societal level but their difficulty in sustaining their family made them feel ashamed, stressed, and ultimately angry. Moreover, some parents reported that the struggle to sustain a livelihood led them to contemplate suicide, as one Syrian father shared, *‘I swear to God, one time...I was going to commit suicide in the centre of Tal Square…because of all the pressure and helplessness I am living in. I’m sick, and I don’t have enough to feed them [children], and what can you do?’* Adolescents described feeling hopeless and suffocated due to perpetual compounding economic difficulties in the community. Both parents and their adolescents described feeling deprived, and this feeling was intensified when they compared themselves to relatives or friends who appeared more financially comfortable.

Another illustration of how community level stressors affected families at the Individual level was highlighted when participants described feeling worried and scared as a result of living in dangerous neighbourhoods. To ensure their safety and wellbeing, they socially isolated themselves and received minimal community support. Women explained that the threatening surrounding environment caused them to feel afraid and to prefer staying at home while their husbands or sons ran errands outside the home. Women’s freedom of movement was limited, particularly without a male escort. A Syrian mother articulated, *‘If I’m on the streets…they berate us…I hear inappropriate talk, filthy talk, directed at us while we walk…young men catcall me, and I get scared for myself…I can’t go out much. I go out less and send my kids instead.’*

The additional stressors facing Syrian families caused them to feel unwanted and powerless. A Syrian mother shared, *‘It’s always like we never ever feel we have worth or value in this country…even if someone hits us, we probably will shut up because we have no one here to support us.’* Discrimination also caused feelings of depression and fear among adolescents, as one Case Manager described, *‘There are adolescents that have depression. The community’s perspective...has caused a fear…a chronic sadness’*. Syrian families opened up about feeling like they did not belong anywhere, they did not have anywhere to go, and they regularly compared their present hardships with the comfortable and happy life they lived in their home country before the war. Many also described experiencing war-related psychological symptoms on a daily basis. After fleeing the war and seeking refuge in another country, Syrian families still felt scared, unsafe, and hopeless.

#### Coping and adapting.

In parallel, at the Individual level, family-based proactive coping emerged, despite the devastating situation facing families. To overcome or adapt to the challenges they were facing and alleviate their difficult situation, families described diverse coping mechanisms and proactive measures. They learned new skills, searched for job opportunities, and utilized their versatile abilities to sustain a livelihood. In addition, when Syrian families were subject to discrimination, they took several measures to protect their children and avoid conflict with others, even moving to another neighbourhood.

To psychologically cope with their hardships, families recognized that their struggles were shared by many. They counted their blessings and expressed gratitude especially when noting their situation was better than others. Religion was an important coping mechanism through tough times as families relied on faith and hope. All families explained that when challenges proved overwhelming and resistant to change, they drew strength from faith-based active patience (or *saber* in Arabic).

Despite the tremendous sources of stress and pain, some participants managed to maintain optimism and determination, as one Lebanese 17-year-old adolescent shared, *‘Nothing is hard for us…we succeed in everything, and we work with everything…I have like 10 professions I can work in.’* Families hoped that children would receive an education in the future if their financial situation improved. Many Syrian families hoped to be given the opportunity to travel to another country. They stated that they dreamed of resettling once again in hopes of a better future, free from discrimination, danger, and poverty, as one 13-year-old Syrian adolescent shared, *‘I like for us to travel. I also dream of traveling for the sake of my sister and her education.’*

### Research team reflexivity

Seven process themes were identified and compiled from interviewer process notes. They include interviewer distress during interviews, participant confusion when being asked about family dynamics, barriers in lengthy consent process, and high social isolation. In addition, themes encompassed differences between interviewers and interviewees, participant over-emphasis of poor living conditions in an effort to receive compensation, and interviewer self-awareness of judgment towards parents with working children. Detailed descriptions of each process theme are provided in S1 Table.

### Community Advisory Board (CAB) feedback on findings

The CABs for this study, made up of at-risk adolescents and their caregivers in two locations, Beddawi and North Beqa’a, were involved in the design of this study and gave feedback on the findings and interpretation. The CABs strongly agreed with the themes that reflected the realities of families’ living situation, in terms of conditions in camps, poor job opportunities, worsening living conditions amidst the economic crisis, little financial and mental health support, unequal access to education, unfair distribution of resources, and poor access to electricity. They emphasised the impact of the economic crisis, calling it ‘a catastrophe.’ They described working from morning to evening, sending their children to work instead of school, and still struggling to meet their family’s basic needs. They also agreed with the psychological impact of the crisis, naming in particular, psychological pressure, exhaustion, anxiety, and devastation.

## Discussion

This study aimed to explore the stressors, and both risk and protective factors of adolescents and their families facing adversity in Lebanon from a systems approach [13]. Findings indicate clear evidence of strengths and challenges to mental health at every systems level within Bronfenbrenner’s social-ecological model [13], demonstrating a complex and multi-directional picture of the social ecology of adolescent and caregiver mental health. At the Individual level, adolescents reported high levels of anxiety, depression, hopelessness, stress, anger, fear and worry as well as loss, grief, psychological pressure, self-harm, and suicidality. They also shared extensive coping and adaptive skills, through values like optimism, determination and hope, active strategies like making ends meet and searching for opportunities, avoidance strategies like avoiding conflict, and faith-based coping like active patience. Within the Microsystem level, different family and gendered roles, such as the peacemaker mother role and the breadwinner father role, impacted adolescent and caregiver mental health, with mothers often emotionally overwhelmed and fathers feeling shame and humiliation when unable to fill the expected role. Healthy and supportive family functioning was also reported across families, with examples of spousal mutual support, close parent-child relationships, and sibling affection. In the Exosystem level, community and neighbourhood factors were identified, including conflict within the wider family and community, social isolation, and restriction of the freedom of adolescents, as well as exposure to discrimination specifically for Syrian refugee families. Within the wider Macrosystem level, stress related to poverty (unemployment and exploitation, and paid and unpaid labour) and access to basic rights, services, and opportunities (education, health and healthcare, aid and services) were identified, impacting on and across all other themes such as adolescent mental health, gendered roles, and family functioning.

Cross-cutting influences of gender and nationality were found across systems levels, in terms of differential experiences of mothers and fathers, and male and female adolescents, as well as differences between Lebanese and Syrian families. This was particularly prominent regarding types of labour and exploitation, family role expectations, and restrictions of freedom of movement and emotional expression in relation to gender, exposure to community hostility, state-level discrimination, and social isolation, in relation to nationality.

These findings, grounded in the lived experiences of adolescents and their families in a protracted humanitarian setting support previous literature on the high levels of psychological distress and mental health difficulties in conflict-affected populations [7], including adolescents in Lebanon [8,9], as well as on the complex links to social determinants of mental health. Findings support the link found previously between armed conflict, displacement [39], poverty [40], exposure to discrimination [41], and poor mental health. In the context of Lebanon in particular, families reported the overwhelming impact of the economic collapse on parent and child mental health, as well as on relationships and family functioning. The devastating impact of the economic crisis described by all families cannot be emphasized enough, including the social and psychological stress of the worsening financial situation, and a heavy sense of uncertainty for the future. Since data collection and at the time of writing, the devaluation of the Lebanese Lira continues to worsen, with the currency having lost over 98% of its value [42]. This has pushed even greater numbers of families into poverty [43], and the economic crisis now ranks among the worst in history [42]. With no functioning government for over two years, a devastating war, and fragile ceasefire, families continue to be exposed to horrendous adversities.

These findings have several implications on the development and implementation of psychosocial support interventions that could bring about significant improvements in supporting adolescent mental health in humanitarian settings. At the Macrosystem level, interventions that do not focus on assumptions of individual and internal errors in thinking or perception, but rather real and unfair social stress and injustice, are, based on our findings, more likely to be relevant and useful to this population. In the context of the region, we found that mental health was commonly referred to as ‘psychological pressure’ (*daghet nafsi* in Arabic), an idiom that much better reflects the external influences of unfairness and structural stress than the term ‘mental health’ does. *Daghet nafsi* is a term widely used in the region [44] and may be a better reflection of systemic and collective distress. Applying Liberation Psychology to our findings, the Cultural Context Model (CCM) helps to link to the wider global struggle of social injustices. CCM, grounded in post-colonial theory, refers to the ways in which institutionalised and social patterns of class, gender, and race shape family conflicts and individual distress [45]. In our context, state-level structural unfairness had a clear impact on both family and adolescent wellbeing, as well as playing out in community dynamics through mechanisms of poverty, neighbourhood disadvantage, and resource competition. Mental health and psychosocial support interventions may therefore need to include a great critical consciousness for these injustices, greater advocacy, and solidarity. In addition, multi-agency coordinated programming can better connect families to services and programs to address holistic needs.

At the Exosystem level, the high level of community isolation and hostility experienced by families was unexpected and of concern, with many families having little to no contact with people outside of their immediate family members, and wide-ranging negative experiences like verbal harassment, physical intimidation, and attempted kidnapping of children. Past research has identified the protective effects of community cohesion [46–48] and social support [49] on the mental health of both adolescents and parents. However, our findings indicate that for adolescents and their families in this study, communities were not serving this function. Interventions could therefore include strengthening community cohesion to improve daily environmental stressors, increase social networks, and support the mobilization of communities to face shared and collective struggles.

At the Microsystem level, findings support previous studies on the buffering effects of positive familial relationships on both adolescent and caregiver mental health [50,51] with many clear examples of support and strength found between and within relationships in the family. Many complex bidirectional challenges to relationships were also identified, contributing to the literature on families’ circular coping in adversity. For example, the impact of adolescents’ externalizing behaviours and anger on caregivers and caregivers’ abilities, as well as the simultaneous impact of caregivers’ own difficulties – such as fathers’ shame and anger over their inability to financially provide for their families, and mothers’ pressure to play the peacemaker role– on adolescents was observed. This is in line with family coercion theory in which cyclical patterns of child behaviour and parenting maintain themselves [52]. Interventions at the family level are therefore warranted to re-formulate and de-construct unhelpful family narratives and roles, strengthen relationships and communication, and generate helpful cyclical interactions of support and nurturing. Such interventions need to reflect the complex nature of families, within cultures and context. There is good evidence for the effectiveness of family-based interventions, including in low resource settings, to reduce adolescent and caregiver distress [30].

Interventions solely addressing individual functioning will do little to address these structural and systemic determinants of mental health, and as advocated in CCM, a greater critical consciousness encompassing these systemic level themes may be better suited to supporting and empowering young people and their families. Greater multi-agency and multi-sector working is also being strongly encouraged through recent efforts by UNICEF, UNHCR and the WHO using the Minimum Services Package (MSP) for Mental Health and Psychosocial Support. In addition to working systemically, results found strong evidence for the faith-based utility of active patience (or *saber* in Arabic). Saber is a term originating in Islam and refers to the balance between tolerating adversity while not giving up [53] – such a concept is of clear value, particularly in the context of protracted conflict, and may integrate well with existing psychological interventions grounded in acceptance and commitment, and mindfulness [54]. Specifically, within interventions, *saber* could be framed as a strength-based resilience tool and can be combined with psychoeducation on emotion regulation skills. The role *saber* plays in enduring adversity without passivity could be emphasized. This faith-based practice can be positioned as complementary to psychosocial goals like mindfulness, acceptance and commitment, and emotion regulation.

### Strengths and limitations

This study benefits from a relatively large sample of at-risk adolescents and their caregivers, including refugee families. It is the first known study to explore social determinants of mental health qualitatively within this population, using a whole-family systems approach.

Nevertheless, this study is limited to one location, and two main nationalities, with limited generalizability to other populations such as Palestinian refugees. Nonetheless, Palestinian families did participate in the CABs that reviewed and validated the findings. Additionally, the data was collected in refugee camps and similar humanitarian settings with limited generalizability to other settings. We also restricted the age group to 12–17 to inform intervention development for this group, so our findings may not be reflective of all adolescent ages. Finally, we faced major challenges in providing compensation, due to the constant minute-by-minute devaluation of the Lebanese Lira. We tried to balance due compensation with avoiding coercion, but we would have liked to provide compensation in USD if it had been possible.

## Conclusions

The findings from this study demonstrate the potential of adolescents, families, and, community to strengthen their mental health and relieve psychological pressure, as well as the societal, structural, and institutional challenges to foster this nurturing environment. Findings have been used to inform the development of a contextually and culturally relevant family-focused psychosocial intervention – the *Sawa Aqwa* (Stronger Together) Family Intervention - that aims to address the complexity and bi-directionality of social-ecological risk factors within families and communities [55].

With the clear evidence of societal, community, and family influences on adolescent mental health, amidst rising mental disorders in this population, humanitarian programming must respond through systemic, holistic, and joined-up programming. Recent efforts to integrate MHPSS across humanitarian sectors [56] can help to achieve this, moving away from siloed care and addressing the wider social-ecological context of adolescents and their caregivers.

## Supporting information

S1 Table Interviewer reflections summary. (DOCX)

S1 Checklist Inclusivity in global research. (DOCX)

## Abbreviations

MHPSS: Mental Health and Psychosocial Support
MENA: Middle East and North Africa
PI: Principal Investigator
COREQ: Consolidated Criteria for Reporting Qualitative Studies
CCM: Cultural Context Model
