# Supplementary material for: The social ecology of adolescent mental health in humanitarian settings: A qualitative study
Source: PLOS Ment Health. 2026 Jan 22;3(1):e0000543. doi: 10.1371/journal.pmen.0000543 (PMC12826506; doi:10.1371/journal.pmen.0000543)
Supplement: S1 Table — (DOCX) [file pmen.0000543.s001.docx]

Supplementary Table 1: Interviewer reflections summary

| **Process theme** | **Description** |
| --- | --- |
| Interviewer distress during interviews | Interviewers experienced distress during interviews due to the unfairness and injustice of participants’ situations, particularly due to the economic collapse and - for Syrian families - structural and state discrimination. Interviewers were at times overwhelmed by the unfairness of the poor housing, living and working conditions of participants, their limited access to health services and education, and the hardships of daily life. Families who clothed their children with materials found in bins, a family living in a garage, the father who tried to make a living selling vegetables but had his cart removed as he did not have a license – these stories were held with a heavy heart, by interviewers but also by those involved in transcribing, translating and analyzing them. The situation in the country (*al wahde*) was often the dominant narrative during interviews, and interviewers often felt concerned that on one hand focusing on the situation made it difficult to get a sense of the emotional and interpersonal experiences within the families, whilst on the other hand diverting the conversation felt insensitive to the severity of the situation. Interviewers commonly felt mixed feelings of sadness, anger and guilt. The desire to help more was also a strong feeling, even with training to handle these scenarios and with clear referral routes. Interviewers were often asked for help financially or for help with resettlement. Whilst great care was taken to establish roles and set expectations before the interviews, requests for help were still commonplace. |
| Family perceptions of being asked about their family dynamics | While our systemic family approach to interviews was helpful to shift focus from individual internal experiences of adolescents on to their wider social system, asking direct questions about interpersonal relationships was either not understood or avoided. Families were often unsure of the meaning of questions about relationship functioning, requiring a more indirect approach using broad and less intrusive questions. Many families would simply reply that their relationships were normal (*a3ede*), and would shift to talking more about tangible difficulties like the situation in the country. Upon reflection and discussion we shifted our approach for follow-up interviews with families to more indirect exploration of interpersonal relationships. This included discussing commonplace challenges and strengths of families they know in general, without directly focusing on their own family. This approach was also suggested by MHPSS facilitator interviewees, who highlighted that direct discussions of family life can be experienced as intrusive and prescriptive, whereas an indirect and suggestive approach is more acceptable. For example, facilitators used the example of psychoeducation being more effective when framed as ‘it is helpful when…’ as opposed to ‘you should.’ |
| Barriers in lengthy consent process | Consent processes were lengthy and individualized, creating some barriers to our whole-family systems interview approach. Families often did not see the necessity of going through the whole consent form, and were trusting of the interviewers motives and use of their data. Interviewers often needed to insist to go over the form in order to ensure informed consent and assent, for many families verbal permission was seen as sufficient and acceptable. Consent processes for family interviews, in which the collective family unit are consenting to participate, was also noted as challenging to an individualized consent and assent process that requires one form per person. Further, and unexpectedly, families were very welcoming of being recorded, and interviewees had the impression this may be due to a desire to have their stories heard and shared, to advocate for external help. Interviewers noted that some families described finding the interview in itself a supportive and helpful discussion. |
| Interviewee high social isolation | Interviewers noted their surprise during interviews of high levels of social isolation as there was an assumption that families would rely on each other through communal, collective, community support. This assumption was directly challenged during interviews, as many families described staying away from others in their communities, daily hostilities, street violence, and neighborhood friction and resentment related to perceptions of unfair distribution of resources by UN agencies. High discrimination against Syrians was noted in particular, including troubling levels of violence exposure in the street – name calling, threats, beatings and even attempted kidnappings. Even in situations where families had some trusted social contacts, extended family or friends, families still described isolation due to not wanting to overburden others, and a preference to keep things within the immediate family. |
| Interviewer and interviewee individual differences | The first interviews were conducted by a Christian Lebanese interviewer, interviewing Muslim Syrian interviewees. They noted a tendency for families to complement Christianity and Lebanon, and to deny or avoid commenting on any possible discrimination or unfair treatment by the Lebanese state. It was felt that this was an attempt by families to be respectful, while the interviewer actually wanted to know what they really thought and tried to create an environment in which these issues could be voiced. Subsequent interviews matched a Syrian Muslim interviewer with Syrian Muslim families, and they noted a clear difference, with families quite open about their socio-political opinions and experiences of the Lebanese state. |
| Interviewee over-emphasizing poor living conditions in an effort to receive compensation | There was potential for biased responding noted due to perceived potential for receipt of financial aid, despite efforts to separate the research from service provision. Facilitators commonly described experiences of families exaggeration of difficulties in order to receive greater support. This was particularly common for facilitators with a background in needs assessments, where an object ‘truth’ of level of need was sought. During family interviews, interviewers noted clear participant preoccupation with receiving financial support. The severity of the living conditions and stressors in daily life were clear for all families – and therefore as a team we consciously took a deliberate stance of not searching for ‘the truth’ of the situation and rather aimed to understand the experience of the family. Our approach aimed to acknowledge that exaggeration or preoccupation is a natural response within a system in which very basic needs are only met through an imperfect assessment of need, and that our interviews were not designed to identify the *need* but the emotional and interpersonal *experience* of the family unit and its members. |
| Interviewer self-awareness of judgement of parents with working children | During the very first interviews, interviewers noted their own assumptions coming to the surface, particularly in relation to parenting and sending children to work instead of school. This was particularly acute for families where fathers were not working, and the challenge was exacerbated by the perceptions described above of ‘exaggerating’ problems. It took extensive discussion and reflection to unpack these assumptions and to be able to emphatically listen and understand the experience of parents, to see past this superficial judgment of the family set up, and to absorb the very different context – albeit within the same country. Interviewers then began to link issues of child labor and poor educational attainment to the much broader and complex issues of working life as a refugee, the high rate of chronic health problems related to living conditions and daily life stressors, but also to understand and empathize with the terrible shame and guilt felt by parents – especially men. This required immense self-reflection, tolerance of difficult emotions and challenges of long-held assumptions, as well as openness to listening, absorbing, and understanding the new perspectives families were communicating. |
